# Pre-Emptive and Non-Pre-Emptive Goal Programming Problems for Optimal Menu Planning in Diet Management of Indian Diabetes Mellitus Patients

**DOI:** 10.3390/ijerph18157842

**Published:** 2021-07-24

**Authors:** Kiran Kumar Paidipati, Hyndhavi Komaragiri, Christophe Chesneau

**Affiliations:** 1 Department of Statistics, Lady Shri Ram College for Women, University of Delhi, New Delhi 110024, India; kirankumar.paidipati@lsr.du.ac.in; 2 Department of Statistics, Pondicherry University, Puducherry 605014, India; hyndhavikomaragiri110@gmail.com; 3 Department of Mathematics, LMNO, Campus II, Université de Caen-Normandie, Science 3, 14032 Caen, France

**Keywords:** diet management, Indian food recipes, diabetes mellitus, linear programming problem (LPP), goal programming problems (GP)

## Abstract

Diet management or caloric restriction for diabetes mellitus patients is essential in order to reduce the disease’s burden. Mathematical programming problems can help in this regard; they have a central role in optimal diet management and in the nutritional balance of food recipes. The present study employed linear optimization models such as linear, pre-emptive, and non-pre-emptive goal programming problems (LPP, PGP and NPGP) to minimize the deviations of over and under achievements of specific nutrients for optimal selection of food menus with various energy (calories) levels. Sixty-two food recipes are considered, all selected because of being commonly available for the Indian population and developed dietary intake for meal planning through optimization models. The results suggest that a variety of Indian food recipes with low glycemic values can be chosen to assist the varying glucose levels (>200 mg/dL) of Indian diabetes patients.

## 1. Introduction

Nutrition is a critical part of health and development. Obviously, better nutrition enhances a strong immune system, lowers the risk of non-communicable diseases, and increases longevity [1]. As an integral part of the nutrition principle, human diet planning is the process of selecting foods or food groups to meet an individual’s nutritional needs [2]. The concept of energy balance and body weight regulation states that when energy intake is greater than energy expenditure, excess calories are stored in the body with a subsequent increase in body weight [3]. It is important to understand and know the optimal requirements of nutrients for good health and well-being with a balanced weight. Many chronic diseases have been directly or indirectly associated with nutritional imbalance. Globally, diabetes mellitus (DM) is the most common non-communicable metabolic disease in which the person has a high blood glucose (blood sugar) level, either due to inadequate insulin production or because the body’s cells do not respond properly to insulin or both [4]. More precisely, DM is a chronic metabolic disease characterized by higher-than-normal blood glucose levels (homeostasis level = 80–120 mg/dL). Glucose levels with measurements such as fasting blood glucose, postprandial blood glucose, and A1c of normal (<100 mg/dL; <140 mg/dL; <5.7%), prediabetes (100–125 mg/dL; 140–200 mg/dL; 5.7–6.4%) and diabetes (>125 mg/dL; >200 mg/dL; >6.4%), respectively.

As an undeniable fact, DM is reaching potentially epidemic proportions in India. The level of morbidity and mortality due to diabetes and its complications is enormous. Many factors influence the prevalence of disease throughout a country [5]. The origin of diabetes in India is multifactorial and includes genetic factors, environmental influences, rising living standards and lifestyle changes. As per 2019 reports of the International Diabetes Federation (IDF), India is one of the topmost countries for the number of people with diabetes from the age group of 20–79 years, where one in six adults with diabetes in the world come from India. Diabetes-related deaths total 1,010,262, with 77 million adults in India at risk. According to a report released by the Ministry of Health and Family Welfare (MoHFW), India in the year 2019, as per the IDF Atlas, India accounts for over 7.29 crore people with diabetes and the estimated increase will be 13.4 crore by 2045. In addition, according to the World Health Organization’s (WHO) Global Diabetes Compact, there has been a 70% increase in global deaths due to diabetes, and it has risen to the ninth leading cause of death in the world. The prediction of people with diagnosed diabetes worldwide will be raised from 420 million to 570 million in 2030 and further it will reach 700 million in 2045.

A three-fold mechanism of proper diet, insulin intervention and physical activity for management of diabetes and slowing down the prevalence of the disease is need of the hour [6]. The prime objective is to maintain a proper diet, i.e., optimizing excessive calories is one of the possible ways to reduce the burden of the disease [7]. Awareness programs to be conducted on diet plans according to the rural and urban parts of India as well as worldwide to enlighten and increase knowledge of planning diabetic diets. Diet optimization for diabetes is increasingly used in the field of nutrition, where it is feasible to achieve all nutritional requirements with the available food recipes [8]. Patients with diabetes are generally put on an approximate intake of a 1200 to 1800 calorie diet per day to promote weight loss and the maintenance of ideal body weight (for diabetic patients having glucose levels of more than 200 mg/dL in the blood).

In this nutritional framework, linear programming problems play a vital role in preparing optimized diet plans with satisfying nutrient requirements. Many researchers have devised various linear and goal programming problems in order to optimize food recipes for global countries in order to provide adequate nutrients for the population. Beginning with the study, to achieve nutritional balance in selected diets by considering 150 raw food materials to satisfy Thais’ daily nutritional requirements with complex inter-relationships of constraints with GP [9]. An efficient method was proposed for solving linear goal programming problems with a smaller number of variables, which is a computationally better optimized model than the traditional methods [10]. Some authors clearly overviewed the techniques such as Pareto efficiency, normalization and non-standard utility function modelling in GP. Further examined the connection between GP and multi-objective programming techniques and utility theory and discussed their ranks as well and another review by the same authors focused on the significant developments of theoretical GP models in the area of intelligent modelling and solution analysis [11,12].

In modern references, some authors proposed a two-stage linear programming model for low-cost healthy diets for Malay households. In Stage-1, the model was formulated to satisfy various nutrient requirements for the pre-determined age-gender groups and Stage-2 focused on optimizing diet plans for the whole household with minimum cost daily diet plans [13]. In addition, other authors proposed the GP nutrition model to minimize the deviations from nutrients, energy value and food cost in order to meet the daily nutrient needs of the reference woman and reference man of households in Bosnia and Herzegovina, developed and extended by proposing a linear programming diet model to maximize energy density for choosing food recipes (containing macro and micro-nutrients) as recommended by WHO in order to reduce food costs [14,15]. Another work was done by proposing linear and goal programming optimization models for analyzing the food basket (consists of nearly 158 general consumption of food recipes) in Bosnia and Herzegovina in order to meet various nutritional requirements of WHO and World Bank recommendations. The parameters such as price and nutrient requirements are linearly related to food weight, where LP models deal with the minimal value and structure of the food basket of an average person and GP models with minimal deviations from nutrient needs if the budget is fixed [16]. Another study proposed an efficient method for solving lexicographic GP problems and its formulation with different variable sizes to be a better model than the existing models. Furthermore, the same researchers reviewed recent improvements and new advancements for solving linear goal programming (LGP) [17,18]. The researchers proposed MinSum, MinMax and extended goal programming problems for diet management problems. The study focused on analyzing the impact of achievement functions in designing diet problems that comply with nutritional, palatable, and cost constraints [19].

Another study proposed a goal programming model for optimal dietary variations for diabetes patients with minimal attainment of sufficient nutrients in Indonesia [20]. Other researchers compared weighted goal programming (WGP) and LP models for the targeted DASH diet’s tolerable intake levels within 1500 mg sodium for various calorie levels. The WGP is more efficient in minimizing the deviation from tolerable target levels at desired cost than the LP model [21]. In parallel, some researchers have developed a nutrition optimization model to satisfy the daily nutrient needs of adolescents through pre-emptive goal programming. To minimize the sum of percentage of nutrient deviations according to its priorities of twenty most frequently consumed foods from Indonesian recommended dietary allowances and the available budget as goal and system constraints [22]. Another study proposed goal programming problems for optimal dietary intake patterns to prevent obesity and calcium deficiency in the Taiwan population. The study concluded with essentially healthier and the promotion of low-fat density foods [23]. The researchers employed linear programming diet optimization models to suggest a realistic and affordable diet with recommended nutrient intake of locally available foods for pregnant women in Malaysia [24]. On the other hand, some authors reviewed four optimization algorithms, such as linear programming, dynamic programming, genetic algorithms and particle swarm optimization, that play a major role in diet optimization problems [25].

All of the studies cited above have focused on optimizing diet plans for both healthy people and those suffering from specific diseases. Limited works have been done on diet plans for diabetes patients based on glucose levels. In the present study, the motivation is to develop six optimized diet plans for diabetes patients with different energy levels (1104 to 1986 Calories). It mainly focuses on optimizing diet plans with varied energy levels (in calories) by satisfying the nutrient requirements of the limited menu recipes for diabetes management in India.

## 2. Materials and Methods

### 2.1. Collection of Data

The nutritional information and dietary intake allowances of nutrients for recipes are collected from the “Nutrify India Now” application (app) authorized by the National Institute of Nutrition (NIN) under the Indian Council for Medical Research (ICMR), India. The food recipes considered based on dietary reference intakes with low glycemic index [26,27,28] are recommended by the NIN, ICMR, India and WHO guidelines for diabetes patients with varying levels of glucose levels (>200 mg/dL). The nutrients such as energy, carbohydrates, protein, fat, saturated fat, fiber, vitamin B2, vitamin B6, iron, zinc, calcium, potassium, sodium, and carotenoids are considered in this study [29]. Six different diet plans with 62 optimal quantities of recipes (70 g of each) are considered for solving optimization problems. The nutrient composition of a diet with various levels of minimal amounts of calories is prepared to optimize the nutrient levels. Optimal diet plans are prepared to serve adequate nutrient compositions through the required quantities of recipes for the diabetes population in India. The list of recipes for different food intervals (Breakfast, Morning/Evening Snacks, and Lunch/Dinner) are represented in Table 1.

### 2.2. Optimization Models for Diet Management of DM Patients

The initial model is derived from the linear programming problem (LPP), with energy (in calories) serving as an objective function and nutrients serving as constraints. Accordingly, to minimize the deviations (over-achievement and under-achievement) of various nutrients from the intended priorities, the preemptive goal programming (PGP) problem was proposed for the achievement of nutrient-oriented goals. Finally, the non-preemptive goal programming (NPGP) model included weights with priorities of nutrient goals to minimize the deviations under the method of weight normalization.

#### 2.2.1. Linear Programming Problem (LPP)

The mathematical model of the problem is written as a linear program, with the objective function corresponding to the total number of energy calories. There are constraints that need to be satisfied regarding nutrient amounts as per various diet plans. Decision variables are based on the type of recipes considered for diabetes intake under study. Thus, we consider:

(1) $$\begin{array}{l} Min\;Z=\sum_{j=1}^{n}E_{jR}\\ Subject\;to\;Constraints\\ \sum_{i=1}^{m}\sum_{j=1}^{n}a_{ij}x_{jR}\leq b_{iNu}\\ \sum_{i=1}^{m}\sum_{j=1}^{n}a_{ij}x_{jR}\geq b_{iNl}\\ x_{jR},b_{iNu},b_{iNl}\geq 0 \end{array}$$

where

E_jR_ = energy values for j number of recipes of various menus.

m = number of nutrients considered in the study (m = 14).

*n* = number of recipes as per diabetes diet plans.

$a_{ij}$ = amount of *i*th nutrient from *j*th recipe.

$b_{iNu}$ = recommended upper level of *i*th nutrient intake.

$b_{iNl}$ = recommended lower level of *i*th nutrient intake.

$x_{jR}$ = decision variables that represent the type of recipes.

#### 2.2.2. Pre-Emptive Goal Programming (PGP) for Optimal Selection of Recipes

Pre-emptive goal programming is a technique which provides a more systematic approach to the problem of balancing the supply of nutrients in a selection of recipes. An objective function is considered to be minimizing deviations from conferring on nutrient priorities. The system and goal constraints (nutrient information in each diet) are equalized to the required limits of nutrient values and the recipes are taken as decision variables. This approach ensures optimum nutritional balance with minimal recipes by satisfying all the constraints.

**Priority 1 (P1): Optimizing Energy Levels**

Goal-1: Minimizing under-achievement and over-achievement of energy (calories).

**Priority 2 (P2): Optimizing the intake of Carbohydrate, Protein, Fat and Saturated Fat**

Goal-2: Minimizing under-achievement and over-achievement of carbohydrate (g).

Goal-3: Minimizing under-achievement and over-achievement of protein (g).

Goal-4: Minimizing under-achievement and over-achievement of fat (g).

Goal-5: Minimizing under-achievement and over-achievement of saturated fat (g).

**Priority 3 (P3): Optimizing the intake of Fiber, Vitamin B_2_ and Vitamin B_6_**

Goal-6: Minimizing under-achievement and over-achievement of fiber (g).

Goal-7: Minimizing under-achievement and over-achievement of vitamin B_2_ (mg).

Goal-8: Minimizing under-achievement and over-achievement of vitamin B_6_ (mg).

**Priority 4 (P4): Optimizing the intake of Iron, Zinc and Calcium**

Goal-9: Minimizing under-achievement and over-achievement of iron (mg).

Goal 10: Minimizing under-achievement and over-achievement of zinc (mg).

Goal 11: Minimizing under-achievement and over-achievement of calcium (mg).

**Priority 5 (P5): Optimizing the intake of Potassium, Sodium and Carotenoids**

Goal 12: Minimizing under-achievement and over-achievement of potassium (mg).

Goal 13: Minimize under-achievement and over-achievement of sodium (mg).

Goal 14: Minimize under-achievement and over-achievement of carotenoids (mg).

The mathematical formulation of the PGP is given by:

(2) $$\begin{array}{l} Min\;Z=\sum_{i=1}^{m}P_{i}(d_{i}^{-}+d_{i}^{+})\\ Subject\;to\;Constraints\\ \sum_{i=1}^{m}\sum_{j=1}^{n}a_{ij}x_{jR}+d_{i}^{-}-d_{i}^{+}=b_{iNr}(System\;Constraints)\\ \sum_{j=1}^{n}E_{jR}+d_{i}^{-}-d_{i}^{+}=b_{iNr}(Goal\;Constraint)\\ x_{jR},b_{iNr},d_{i}^{-},d_{i}^{+}\geq 0 \end{array}$$

where

E_jR_ = energy values for j number of recipes of various menus.

m = number of nutrients considered in the study (m = 14).

*n* = number of recipes as per diabetes diet plans.

$a_{ij}$ = amount of *i*th nutrient from *j*th recipe.

$b_{iNr}$ = recommended required level of *i*th nutrient intake.

$x_{jR}$ = decision variables that represent the type of recipes.

*P*_i_ = specified priorities of nutrients.

$d_{i}^{-},d_{i}^{+}$ are the under-achievement and over-achievement of each nutrient.

#### 2.2.3. Non-Pre-Emptive Goal Programming (NPGP) for Optimal Selection of Recipes

In NPGP, minimizing the ratio of sum of deviations of nutrient goals with priorities and weights of required nutrient levels by percentage normalization method. The coefficients for deviational variables in the objective function reflect the importance and desirability of the deviations from various nutrient goals. The constraints and decision variable information are identical to those found in PGP. Thus, we consider:

(3) $$\begin{array}{l} Min\;Z=\sum_{i=1}^{m}P_{i}(d_{i}^{-}+d_{i}^{+})/b_{iNr}\\ Subject\;to\;Constraints\\ \sum_{i=1}^{m}\sum_{j=1}^{n}a_{ij}x_{jR}+d_{i}^{-}-d_{i}^{+}=b_{iNr}(System\;Constraints)\\ \sum_{j=1}^{n}E_{jR}+d_{i}^{-}-d_{i}^{+}=b_{iNr}(Goal\;Constraint)\\ x_{jR},b_{iNr},d_{i}^{-},d_{i}^{+}\geq 0 \end{array}$$

where

E_jR_ = energy values for j number of recipes of various menus.

m = number of nutrients considered in the study (m = 14).

*n* = number of recipes as per diabetes diet plans.

$a_{ij}$ = amount of *i*th nutrient from *j*th recipe.

$b_{iNr}$ = recommended required level of *i*th nutrient intake.

$x_{jR}$ = decision variables that represent the type of recipes.

*P*_i_ = specified priorities of nutrients.

$d_{i}^{-},d_{i}^{+}$ are the under-achievement and over-achievement of each nutrient.

## 3. Results and Discussion

This section explores the solutions of six optimized diets for satisfying the nutrient requirements through LPP, PGP and NPGP which are represented in Table 2, Table 3, Table 4, Table 5, Table 6 and Table 7.

**Figure 1:**
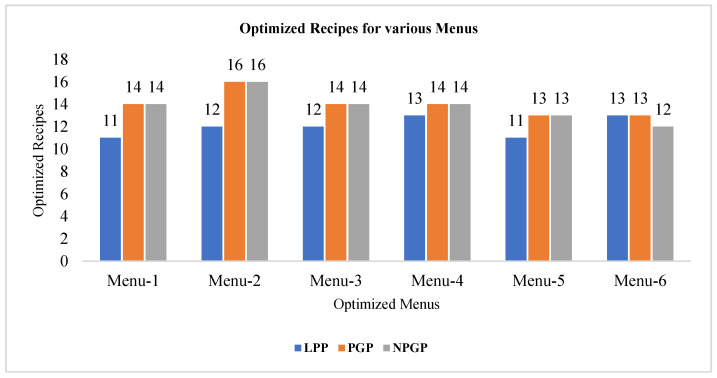
Total number of optimized recipes for various Menus through LPP, GPP, and NPGP.

Overall, the results show diet plans with optimal food recipes for satisfying the nutritional requirements of Menus 1–6 with five-time intervals (breakfast, morning snack, lunch, evening snack, and dinner) for the total energy levels such as 2199.9, 2310.8, 1785.7, 2606, 2196.2, and 2616.8 calories which are optimized by LPP, PGP and NPGP as shown in Table 2, Table 3, Table 4, Table 5, Table 6 and Table 7. Menu-1 framed with 16 recipes and the solution of LPP, optimized 11 recipes with energy levels to 1535 calories. Further, the solution of PGP and NPGP optimized 14 recipes for energy levels of 1570 calories. Menu-2 contains 16 recipes and the results of LPP optimized 12 recipes with energy levels of 1520.9 calories and the solutions of PGP and NPGP have considered all 16 food recipes with 1756.7 calories of energy out of 2310.8 calories. Menu-3 consists of 16 recipes and the LPP solution optimized 12 recipes with energy levels of 1272.5 calories and solutions of PGP and NPGP have considered 14 food recipes with 1284 calories of energy out of 1785.7 calories. Furthermore, Menu-4 with 16 recipes and the solution of LPP optimized 13 recipes with energy levels of 1568 calories and solutions of PGP and NPGP optimized 14 recipes with 1583.7 calories of energy out of 2606 calories. Menu-5 with 13 recipes and the solution of LPP optimized 11 recipes with energy levels of 1096.4 calories and the solutions of PGP and NPGP optimized all 13 recipes with 1104.7 calories of energy out of 2196.2 calories. Finally, Menu-6 with 16 recipes and the solution of LPP optimized 13 recipes with energy levels of 1962.9 calories and the solutions of PGP and NPGP optimized 13 and 12 recipes with 1986.8 calories of energy out of 2616.8 calories. Hence, LPP, PGP and NPGP optimized the entire menu by minimizing the total energy for meeting the sufficient nutritional requirements of patients with blood glucose levels of above 200 mg/dL which are depicted in Table 8. The menus with optimized recipes obtained through LPP, PGP and NPGP are represented in Figure 1.

Nutritional recommendations for optimal disease management are challenging to implement in reality. Patients with DM are already overweight at diagnosis and gain more weight while taking oral medications and/or insulin. DM individuals may also increase energy intake through possible overtreatment of medication-induced hypoglycemia [3]. When tailoring dietary intake levels, the priority should be given to energy reduction and changing dietary composition to optimize intake of essential nutrients and maintain eating patterns. The present study focuses on choosing food recipes with a low glycemic index with the respective nutritional values [30,31]. The linear and goal programming problems in the study produce optimal and feasible solutions to the diet problems in balancing nutrient requirements. Goal programming problems are more flexible in producing a combination of food recipes to fulfill all nutrient goals according to their priorities. The results obtained from PGP and NPGP meet the daily nutrient portions in menu planning. However, alternative menu combinations are possible to replace food recipes with the same source of nutrients through these optimization models. Quantities of food recipes can be selected based on the varying sugar levels of diabetes patients for satisfying energy balance. The study helps to maintain glucose levels with optimized food menus and to promote a healthy lifestyle for DM patients in India.

## 4. Conclusions

The study mainly focused on developing pre-emptive and non-pre-emptive goal programming problems in the optimal selection of menu planning for diabetes patients in India. It has explored various menus with different energy levels that satisfy sufficient nutrient requirements for the glucose levels of individuals (>200 mg/dL). The goal programming methods optimized the energy levels (calories) by considering maximum allocation of food recipe quantities and minimized the overall deviations of over and under-achievements of nutrient goals. Efforts are also made to ensure food recipes that contain a low percentage of carbohydrates, fats, and satisfy all of the other required nutrients for diabetes patients, which plays an important role in menu selection. The computed optimal menus are very helpful for varying glucose levels of diabetes patients as they are within the suggested diabetic intake range. The study helps to select food recipes that are widely available to the extensive public and to reduce the economic burden of the Indian population.

### Limitations of the Study

The optimal selection of food recipes is limited to normal diabetes patients with varying glucose levels (>200 mg/dL). The study can be extended to select optimal food recipes for specific complications such as gestational diabetes, retinopathy, nephropathy and diabetic foot ulcers, etc., and also other diseases (i.e., hypertension, eczema, cardiovascular and other chronic diseases) through the proposed optimization models. Our study is limited to employing pre-emptive and non-pre-emptive goal programming problems, which can be improved by utilizing broader areas in optimization techniques for yielding more accurate results to the disease specific. The future scope of GP models can be extended by incorporating various types of normalization procedures, introducing utility theories and solution analysis to develop precise diet plans for disease specific.

## Figures and Tables

**Table 1:** List of food recipes.

Breakfast	Morning/Evening Snacks	Lunch/Dinner
Idli, Groundnut Chutney, Wheat Bread, Omlet, Upma, Tomato Chutney, Vermicelli Upma, Coriander Chutney, Oat Meal	Coffee, Keera, Sour Soup, Basin-ki-Burfi, Tea, Orange, Marie Biscuit, Taco Salad, Pulka, Carrot and Cauliflower Curry, Guava, Orange Juice, Sandwich, Green Tea, Green Apple, Salt Biscuits, Grapes, Vegetable Salad, Lime Juice, Papaya, Salt Biscuits, Fruit Cake	Cooked Rice, Boiled Egg, Red Gram Dhal, Brinjal Curry, Sambhar, Yogurt, Roti, Vegetable Kurma, Buttermilk, Brown Rice, Fish Fillet, Lentil Dhal, Amarnath Curry, Korralu, Stew (Mutton with Vegetables), Green Gram Dhal, Roasted Dhal Chutney, Chapati, Ridge Gourd Curry, Bengal Gram Dhal, Rasam, Chicken Curry, Spinach Dhal, Lady’s Finger Curry, Cabbage Curry, Skimmed Milk, Brown Rice, Fish Curry, Roasted Dhal Chutney, Greenleaf Curry, Mixed Vegetable Curry.

**Table 2:** Optimal Food Recipes (in grams) of Menu-1 with Total Energy (2199.9 Calories).

Recipe/Time Intervals	LPP	PGP	NPGP
**Breakfast**			
Idli	-	50.9	35.7
Groundnut Chutney	68.8	99.3	69.5
Coffee	-	-	-
**Morning Snack**			
Keera	189.7	191.7	134.2
Sour Soup	15.7	71.7	50.2
**Lunch**			
Cooked rice	123.2	164.3	115
Boiled Egg	83.3	103.9	72.8
Red Gram Dal	-	71	49.7
Brinjal Curry	56.7	83.5	58.5
Sambar	156.8	145.9	102.1
Yogurt	-	30.8	21.6
**Evening Snack**			
Basin-ki-Burfi	59	89.6	62.7
Tea	406.7	49.7	348.1
**Dinner**			
Roti	91	119.1	83.4
Vegetable Kurma	35.7	83.7	58.6
Buttermilk	-	-	-
**Optimized Calories**	**1535**	**1570**	

**Table 3:** Optimal Food Recipes (in grams) of Menu-2 with Total Energy (2310.8 Calories).

Recipe/Time Intervals	LPP	PGP	NPGP
**Breakfast**			
Wheat Bread	-	95.3	66.724
Omlet	7.8	101.0	70.714
Coffee	197	75.1	52.6
**Morning Snack**			
Orange	47.3	107	74.9
Marie Biscuit	64.1	105	73.5
**Lunch**			
Brown rice	51.2	95.9	66.8
Fish fillet	69.4	100	70
Lentil Dal	172.5	99.5	69.7
Amarnath Curry	108.7	93.8	69.9
Buttermilk	-	127.1	89
**Evening Snack**			
Taco Salad	-	117.1	82
Tea	34.9	113.4	79.4
**Dinner**			
Pulka	123.9	97.7	68.4
Carrot and Cauliflower Curry	-	91.7	64.2
Yogurt	60.7	99.5	69.7
**Optimized Calories**	**1520.9**	**1756.7**	

**Table 4:** Optimal Food Recipes (in grams) of Menu-3 with Total Energy (1785.7 Calories).

Recipe/Time Intervals	LPP	PGP	NPGP
**Breakfast**			
Upma	79.6	152.8	106.9
Tomato Chutney	-	127.4	89.2
Tea	-	76.5	53.6
**Morning Snack**			
Guava	89.9	117.1	82
Orange Juice	229.1	80.3	56.2
**Lunch**			
Korralu	57.1	119.9	84
Stew (mutton with vegetables)	26.6	129.8	90.9
Green dal	95.6	86.9	60. 9
Roasted Dal Chutney	38.2	107.5	75.3
Rasam	238.3	184	128.8
Yogurt	141.9	114.5	80.2
**Evening Snack**			
Sandwich	94.5	74.3	52
Green Tea	-	-	-
**Dinner**			
Chapathi	69.3	69	48.4
Ridge Gourd curry	-	-	-
Buttermilk	45.9	14.9	9.9
**Optimized Calories**	**1272.5**	**1284.0**	

**Table 5:** Optimal Food Recipes (in grams) of Menu-4 with Total Energy (2606 Calories).

Recipe/Time Intervals	LPP	PGP	NPGP
**Breakfast**			
Idli	161.1	130.2	91.1
Groundnut Chutney	70.1	98.8	69.2
Coffee	100.3	-	-
**Morning Snack**			
Green Apple	244.5	116.5	81.5
Salt Biscuits	22.3	100	70.2
**Lunch**			
Cooked rice	103. 5	44.4	31.1
Ridge Gourd Curry	-	170.2	119.2
Omlet	78. 7	101.3	70.9
Dhal (veg)	107.3	56	39. 3
Rasam	177.3	-	-
Yogurt	86.8	80.7	56.5
**Evening Snack**			
Sandwich	73.6	113.1	79.2
Tea	-	350.6	245.4
**Dinner**			
Roti	44.5	104.9	73.5
Carrot and Cauliflower Curry	-	91.6	64.1
Buttermilk	56.7	70.2	49
**Optimized Calories**	**1568**		**1583.7**

**Table 6:** Optimal Food Recipes (in grams) of Menu-5 with Total Energy (2196.2 Calories).

Recipe/Time Intervals	LPP	PGP	NPGP
**Breakfast**			
Vermicelli Upma	107.3	101.2	65.8
Coriander Chutney	73.2	89.9	62
**Morning Snack**			
Grapes	74.8	98.1	65.7
**Lunch**			
Korralu	149.3	96.4	64.7
Chicken Curry	113.0	102.4	73.6
Spinach Dal	29.9	46.3	34.1
Lady’s finger Curry	-	58.4	38.6
Yogurt	128.1	116.9	77
**Evening Snack**			
Vegetable Salad	137.1	121.7	80
Green Tea	32.6	15.2	18.4
**Dinner**			
Chapati	135.8	31.4	225.2
Cabbage Curry	-	105	74.2
Skimmed milk	24.3	171.9	129
**Optimized Calories**	**1096.4**		**1104.7**

**Table 7:** Optimal Food Recipes (in grams) of Menu-6 with Total Energy (2616.8 Calories).

Recipe/Time Intervals	LPP	PGP	NPGP
**Breakfast**			
Oatmeal	74	81.6	57.1
Boiled egg	59	105.3	73.7
Lime juice	-	93.7	65.6
**Morning Snack**			
Papaya	54.1	112.2	101
Salt Biscuits	83.9	129.1	90.4
**Lunch**			
Brown rice	58.2	94.1	65.9
Fish	79.4	119.8	83.9
Roasted dhal chutney	69.2	100	70.3
Green leafy curry	-	-	-
Rasam	110.8	82.4	-
Yogurt	121.4	-	-
**Evening Snack**			
Fruit cake	62.9	73	51.1
Coffee	49.6	192.7	134.9
**Dinner**			
Roti	76.8	110.7	77.5
Mixed vegetable curry	90.9	140.8	98.6
Buttermilk	-	-	-
**Optimized Calories**	**1962.9**		**1986.8**

**Table 8:** Menus Information of Total and Optimized Calories.

Number of Menus	Total Calories	Optimized Calories	
		LPP	PGP/NPGP
Menu-1	2199.9	1535	1570
Menu-2	2310.8	1520.9	1756.7
Menu-3	1785.7	1272.5	1284.0
Menu-4	2606	1568	1583.7
Menu-5	2196.2	1096.4	1104.7
Menu-6	2616.8	1962.9	1986.8

## Data Availability

The data presented in this study are available on request from the corresponding author.
